# Multi-Modal Homing in Sea Turtles: Modeling Dual Use of Geomagnetic and Chemical Cues in Island-Finding

**DOI:** 10.3389/fnbeh.2016.00019

**Published:** 2016-02-22

**Authors:** Courtney S. Endres, Nathan F. Putman, David A. Ernst, Jessica A. Kurth, Catherine M. F. Lohmann, Kenneth J. Lohmann

**Affiliations:** Biology Department, University of North CarolinaChapel Hill, NC, USA

**Keywords:** navigation, magnetism, sea turtles, homing, olfaction

## Abstract

Sea turtles are capable of navigating across large expanses of ocean to arrive at remote islands for nesting, but how they do so has remained enigmatic. An interesting example involves green turtles (*Chelonia mydas*) that nest on Ascension Island, a tiny land mass located approximately 2000 km from the turtles’ foraging grounds along the coast of Brazil. Sensory cues that turtles are known to detect, and which might hypothetically be used to help locate Ascension Island, include the geomagnetic field, airborne odorants, and waterborne odorants. One possibility is that turtles use magnetic cues to arrive in the vicinity of the island, then use chemical cues to pinpoint its location. As a first step toward investigating this hypothesis, we used oceanic, atmospheric, and geomagnetic models to assess whether magnetic and chemical cues might plausibly be used by turtles to locate Ascension Island. Results suggest that waterborne and airborne odorants alone are insufficient to guide turtles from Brazil to Ascension, but might permit localization of the island once turtles arrive in its vicinity. By contrast, magnetic cues might lead turtles into the vicinity of the island, but would not typically permit its localization because the field shifts gradually over time. Simulations reveal, however, that the sequential use of magnetic and chemical cues can potentially provide a robust navigational strategy for locating Ascension Island. Specifically, one strategy that appears viable is following a magnetic isoline into the vicinity of Ascension Island until an odor plume emanating from the island is encountered, after which turtles might either: (1) initiate a search strategy; or (2) follow the plume to its island source. These findings are consistent with the hypothesis that sea turtles, and perhaps other marine animals, use a multi-modal navigational strategy for locating remote islands.

## Introduction

Diverse marine animals, including fishes (Svedäng et al., 2007; Rooker et al., 2008), reptiles (Allard et al., 1994), and mammals (Hoffman and Forcada, 2012), migrate long distances through the open sea to arrive at specific locations where they reproduce. How animals navigate through the open ocean, and how they find and recognize specific reproductive areas, has remained enigmatic for decades (Carr, 1967; Harden-Jones, 1968; Lohmann et al., 1999, 2013).

Among marine migrants, sea turtles are particularly interesting subjects for navigational studies, not only because they often migrate across long distances to nest at specific locations, but because some populations nest on continental coastlines while others nest on islands. Recent studies on loggerhead turtles that nest along the southeastern U.S. coast have provided evidence that such turtles exploit Earth’s magnetic field when navigating to their coastal nesting areas (Brothers and Lohmann, 2015). Specifically, turtles derive long-distance navigational information by detecting the magnetic intensity and inclination angle (the angle at which field lines intersect Earth’s surface; Lohmann and Lohmann, 1994, 1996; Putman et al., 2011; Lohmann et al., 2012). These parameters vary predictably across the globe (Gould, 1982; Skiles, 1985). As a result, each area of coastline is typically marked by a different isoline of inclination and a different isoline of intensity and thus has a unique magnetic signature (Lohmann et al., 2008b). Growing evidence indicates that sea turtles (Lohmann et al., 2004; Putman and Lohmann, 2008; Brothers and Lohmann, 2015), as well as salmon (Bracis and Anderson, 2012; Putman et al., 2013, 2014), return to specific areas along continental coastlines by recognizing magnetic signatures that exist at or near the target area.

Some populations of sea turtles, however, nest on islands instead of on continental beaches. For such turtles, the process of locating an island and nesting area may require more than the geomagnetic field alone (Lohmann et al., 1999, 2008a). At islands, strategies of magnetic navigation are complicated by two factors: (1) the target is considerably smaller and easier to miss than a continental coastline; and (2) Earth’s field changes gradually over time (Lohmann et al., 1999, 2008a). Along continental coasts, the field change typically causes the magnetic signature at a given location to move along the shoreline to an adjacent area of beach (Lohmann et al., 2008b). In contrast, the magnetic signature that exists at a small island often moves offshore into the open sea (Lohmann et al., 1999). For this reason, magnetic navigation alone appears insufficient to explain island-finding in sea turtles. Instead, it has been hypothesized that turtles use multiple sensory cues to locate an island nesting beach (Lohmann et al., 1999, 2008a).

A classic example of island-nesting sea turtles exists at Ascension Island, a small (5 km diameter) island in the South Atlantic that serves as a rookery for thousands of green turtles (Carr, 1975; Mortimer and Carr, 1987; Godley et al., 2001). Most or all of these turtles migrate to Ascension from feeding grounds along the coast of Brazil, a distance exceeding 2000 km. The mechanisms that underlie long-distance navigation to Ascension remain unknown, but one hypothesis is that magnetic cues guide turtles into the vicinity of the island, after which chemical cues, perhaps in combination with search patterns, are used to localize the island (Lohmann et al., 2008a).

As a first step toward investigating this hypothesis, we used oceanic, atmospheric, and geomagnetic models to assess whether magnetic and chemical cues might plausibly allow turtles to locate Ascension Island. The results of simulations suggest that magnetic cues alone or chemical cues alone are insufficient to guide turtles to Ascension, but sequential use of the two might provide a reliable strategy for locating the island.

## Materials and Methods

### Modeling Geomagnetic Drift

At Ascension Island, adult female turtles typically return every 2–5 years to nest, while spending the intervening years at distant feeding grounds (Carr, 1975; Mortimer and Carr, 1987; Mortimer and Poirtier, 1989). One question of interest is thus how much the magnetic field at Ascension Island changes during a 2–5 year absence, and what impact this might have on magnetic navigation strategies (Lohmann et al., 1999). In addition, turtles undertaking their first reproductive migration to the island have probably been absent from the island for considerably longer than 5 years. Although the age at which turtles mature is not known for Ascension Island green turtles, it has been estimated at 25 years for other green turtle populations (Frazer and Ehrhart, 1985; Frazer and Ladner, 1986).

**Figure 1:**
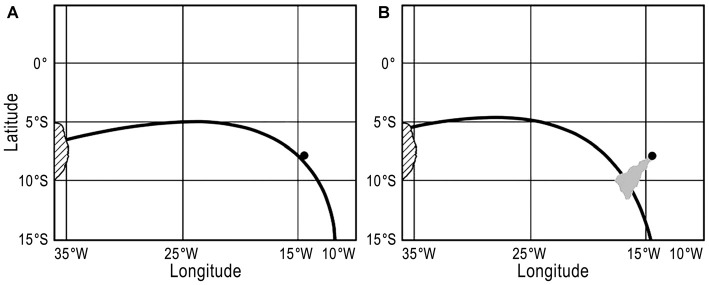
**(A)** A map depicting a magnetic isoline that runs from Brazil to Ascension Island. Brazil is on the left, marked by hash marks, while Ascension Island is the small black dot. The black line represents the magnetic intensity isoline that intersected both Ascension Island and Brazil in 1985. **(B)** Map as depicted in **(A)** but 25 years later, in 2010. The same isoline shown in **(A)**, which intersected both Ascension and Brazil in 1985, has now shifted so that it no longer intersects Ascension Island but instead runs south of the island. However, a waterborne odor plume, depicted in gray, emanates from the island and intersects the intensity isoline. Thus, if turtles swim along the isoline in the later stages of their migration (or as part of a search strategy), then they might plausibly encounter a plume of waterborne odorants that could lead them to the island.

To investigate changes in Earth’s magnetic field and how they might affect turtle navigation, we used the International Geomagnetic Reference Field (IGRF-10; MacMillan and Maus, 2005) to estimate values of magnetic field intensity at the center of Ascension Island (7.933°S, 14.367°W). As in Putman and Lohmann (2008), these values were determined at 5-year intervals from 1900 to 2010. To examine the field change that would occur for a first-time migrant returning to the island after an absence of 25 years, we then plotted the values of the original intensity isoline 25 years later (see Figures 1A,B). In other words, a map was generated which showed the location, in 1925, of the isoline for magnetic intensity that had previously existed at Ascension Island in 1900; similarly, the isoline that existed at Ascension Island in 1905 was plotted in the position it had moved to by 1930, and so on. Similar maps were generated assuming that turtles returned after 5 years, as the case would be for a remigrating adult that had learned the intensity at Ascension Island during its last reproductive effort.

Although turtles can detect both field intensity and inclination (Lohmann and Lohmann, 1994, 1996), we focused on intensity because analyses revealed that, during the last century, isolines of intensity near Ascension Island have tended to drift less than have isolines of inclination (Endres, unpublished data). We therefore chose the global magnetic parameter that appears most stable and favorable for helping turtles migrating from Brazil reach the vicinity of the island, with a view toward assessing whether a simple navigational strategy involving swimming along an isoline (Lohmann et al., 2007), combined with chemical cues, might be sufficient for reaching the target.

### Modeling Odorant Dispersal

Sea turtles are known to detect both waterborne (Manton et al., 1972; Grassman and Owens, 1982; Grassman et al., 1984; Constantino and Salmon, 2003; Piovano et al., 2004) and airborne (Endres et al., 2009; Endres and Lohmann, 2012, 2013) odorants. The dispersal of waterborne odorants was simulated using the particle-tracking program ICHTHYOP v. 2 (Lett et al., 2008) and the Global Hybrid Coordinate Ocean Model (HYCOM; Bleck, 2002). We used Global HYCOM output with a spatial resolution of 0.08° (~8–9 km) and a snapshot of ocean velocity at 00:00 h GMT (Putman and He, 2013). HYCOM uses data assimilation to produce “hindcast” model output that reflects *in situ* and satellite measurements. Global HYCOM thus resolves mesoscale processes such as meandering currents, fronts, filaments, and oceanic eddies (Bleck, 2002; Chassignet et al., 2006). For advection of particles through HYCOM velocity fields, ICHTHYOP implemented a Runge Kutta 4th order time-stepping method (Lett et al., 2008). Additionally, we included horizontal dispersion in simulations to account for turbulent sub-gridscale processes not characterized by HCYOM (for details, see Lett et al., 2008). This modeling approach yields predictions of transport broadly consistent with the tracks of drifting (Lagrangian) buoys (see Putman and He, 2013; Putman and Mansfield, 2015) and we used it here to characterize the movement of water in the vicinity of Ascension Island, implicitly assuming that the hypothetical odorant(s) would be dispersed accordingly (Putman et al., 2014).

In the present case, our goal was not to track the oceanic conditions at a specific time, but to realistically depict typical oceanic variability during intervals when adult green turtles are homing to Ascension Island. Virtual particles were randomly seeded within an area (10 km × 10 km zone) centered on Ascension Island. Particles were tracked at five vertical layers spanning the depths over which turtles migrate (0, 10, 20, 30, and 50 m from the surface; Hays et al., 2001). From December 15 to April 29 (the duration of the main nesting season at Ascension Island; Godley et al., 2001), 100 particles were released at each of the five vertical layers every day. We performed a sensitivity analysis in which we assumed the hypothetical odorants being dispersed by currents maintained their integrity for 15, 30, or 45 days, after which they were removed from the simulation. Simulations were performed for multiple years (2004, 2005, 2006, and 2007) to account for annual variability in ocean conditions.

A similar approach was used to model the dispersal of airborne particles. Virtual particles were released every hour from a height of 0 meters at the center of Ascension Island and tracked for 48 h using the Hybrid Single-Particle Longrarian Integrated Trajectory (HYSPLIT) model (Draxler and Hess, 1997, 1998; Draxler, 1999). We deemed 48 h to be a conservative measure of how long airborne odorants associated with land might last; estimates of long-range transport of a variety of organic compounds range from 6 days to many years (Cousins et al., 2003). To capture annual variation in winds during the green turtle nesting season at Ascension Island, particles were released between December 15 and April 29 for the years 2009, 2010, and 2011.

### Analytical Approach

**Figure 2:**
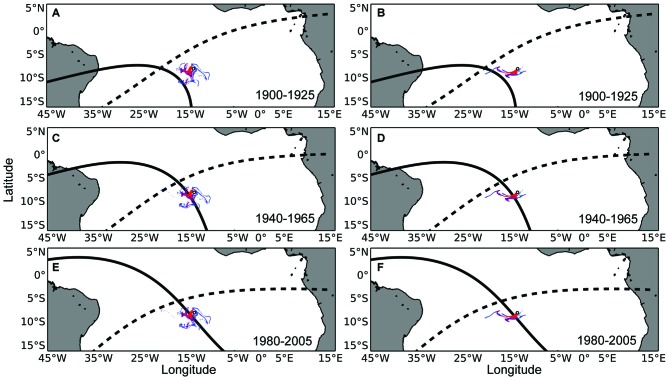
**Maps of waterborne odor plumes in combination with the isolines of magnetic intensity and inclination angle at Ascension Island. (A)** The small white circle represents Ascension Island. The intensity (solid black) and inclination angle (dashed black) isolines that existed at Ascension Island in the year 1900 have been plotted in the year 1925, when turtles that left the island in 1900 (as hatchlings) would be expected to return for the first time to mate and nest (as adults). Colored swirls emanating from the island represent the dispersal of waterborne odorant particles within the top 50 m of the ocean surface at the beginning of the nesting season. Red, purple, and blue swirls represent odors that persist in the environment for 15, 30, and 45-day respectively. **(B)** Same as in **(A)**, but with simulated odorants released towards the end of the nesting season. **(C,D)** Same as in **(A,B)**, but with magnetic isolines associated with Ascension Island in 1940 plotted 25 years later, in 1965. **(E,F)** Same as in **(A,B)**, but with magnetic isolines associated with Ascension Island in 1980 plotted 25 years later, in 2005. Hypothetically, a turtle returning to Ascension Island after a 25 year absence might follow the intensity isoline on which it imprinted as a hatchling (Lohmann et al., 2008a; Brothers and Lohmann, 2015) to arrive in the vicinity of the island, where it would then detect olfactory cues which might guide it the rest of the way to the island.

**Figure 3:**
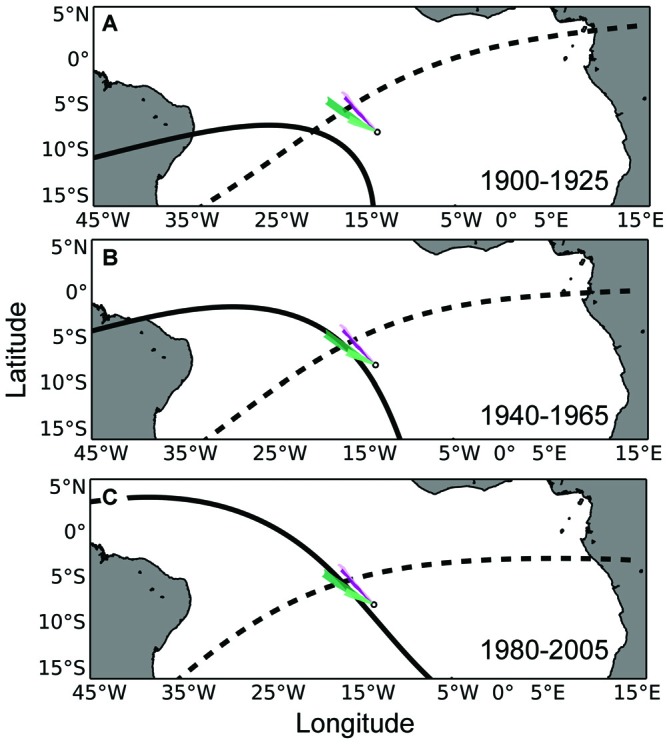
**Maps of airborne odor plumes in combination with the isolines of magnetic intensity and inclination angle at Ascension Island. (A)** The small white circle represents Ascension Island. The intensity (solid black) and inclination angle (dashed black) isolines that existed at Ascension Island in the year 1900 have been plotted in the year 1925, when turtles that left the island in 1900 (as hatchlings) would be expected to return for the first time to mate and nest (as adults). Colored plumes emanating from the island represent the dispersal of airborne odorant particles for a duration of 48 h. Purple-colored and green-colored plumes represent simulations toward the beginning and end of the nesting season, respectively (lighter colors indicate simulations from the same year). **(B)** Same as in **(A)**, but with magnetic isolines associated with Ascension Island in 1940 plotted 25 years later, in 1965. **(C)** Same as in **(A)**, but with magnetic isolines associated with Ascension Island in 1980 plotted 25 years later, in 2005. Similar to waterborne cues (Figure 2), airborne cues might enlarge the homing target for a turtle initially using the geomagnetic field to return to the vicinity of its natal site.

We plotted snapshots of the simulated waterborne odor plumes emanating from the island at 10 evenly-spaced periods during each year, resulting in 40 odor plumes per condition. The 40 odor plumes were overlaid on maps of geomagnetic drift (depicting 25 and 5 years of field drift; see Figure 2). We then assessed the frequency with which simulated plumes intersected the isolines previously associated with Ascension Island to determine whether such plumes could provide a reliable “second coordinate” by which turtles could localize the island. A similar analysis was performed for the airborne odorants. These plumes were plotted at five evenly-spaced periods during each year, resulting in a total of 15 plumes. These plumes were also plotted over each map of geomagnetic drift to assess whether airborne odor plumes could be used as a secondary cue by turtles to relocate Ascension Island (see Figure 3).

## Results

Maps depicting intensity isolines in combination with simulated waterborne odor plumes from Ascension Island showed a high percentage of overlap between these two potential cues. This finding lends credence to the possibility that a turtle migrating to the island from Brazil might be able to follow an intensity isoline into the vicinity of Ascension Island, and then use waterborne chemical cues to find the island directly.

**Figure 4:**
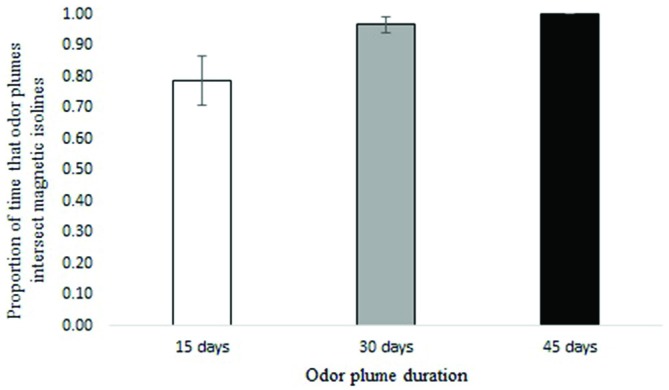
**Proportion of time that particles intersect the 25-year intensity isoline for 15, 30 and 45-day odorant durations (averages from years 2004–2007).** Error bars represent 95% confidence intervals.

**Figure 5:**
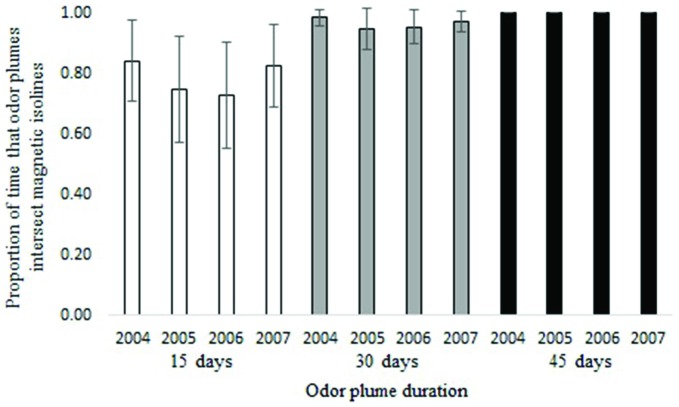
**Proportion of time that particles intersect the 25-year intensity isoline for 15, 30 and 45-day odorant durations (yearly average).** Error bars represent 95% confidence intervals.

Waterborne odor plumes intersected 25-year isolines between 72 and 100 percent of the time and 5-year isolines nearly 100 percent of the time, depending on the duration of the odorant. Across all years, odor plumes lasting 45 days contacted 25-year isolines 100 percent of the time; odor plumes lasting 30 days contacted isolines 97 percent of the time on average, and odor plumes lasting 15 days did so 78 percent of the time on average (Figure 4). Variation in ocean circulation among different years had a greater influence on odor plumes intersecting the intensity isoline when odorant durations were shorter (Figure 5).

**Figure 6:**
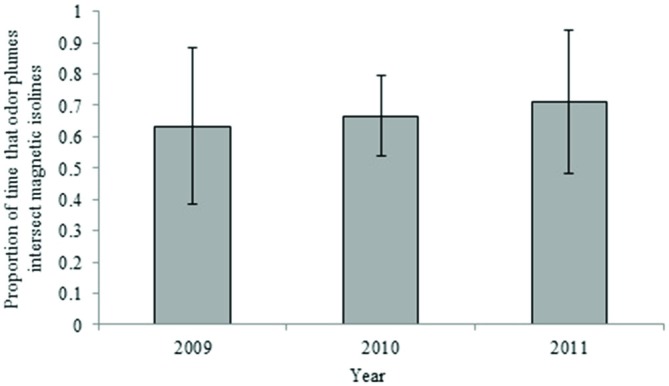
**Proportion of time that airborne odor particles intersect the 25-year intensity isoline (yearly averages).** Error bars represent 95% confidence intervals.

**Figure 7:**
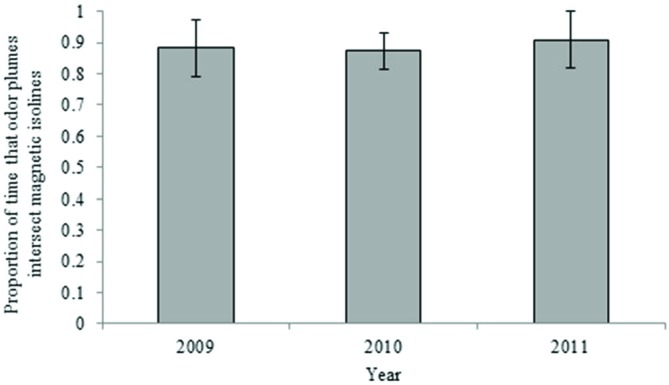
**Proportion of time that airborne odor particles intersect the 5-year intensity isoline (yearly averages).** Error bars represent 95% confidence intervals.

Maps depicting intensity isolines in combination with simulated airborne odor plumes showed a lesser, but still frequent, overlap between these two parameters over the last century. Odor plumes intersected 25-year isolines between 63 and 71 percent of the time, depending on atmospheric conditions in a given year (Figure 6). Airborne plumes came into contact with 5-year isolines between 82 and 91 percent of the time (Figure 7).

**Figure 8:**
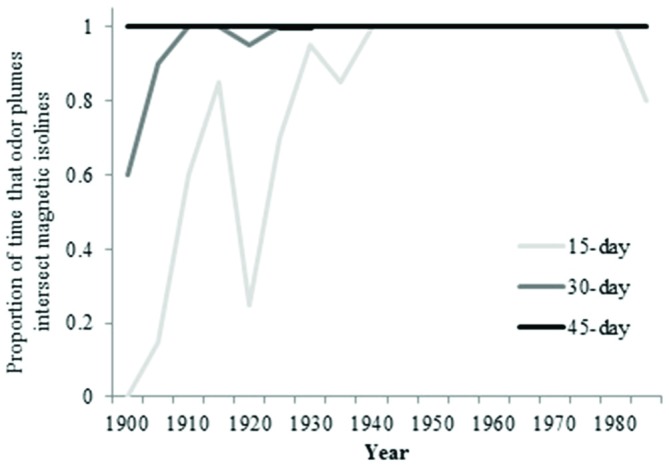
**Proportion of time that particles intersect 25-year intensity isoline (4-year average) over the past century**.

**Figure 9:**
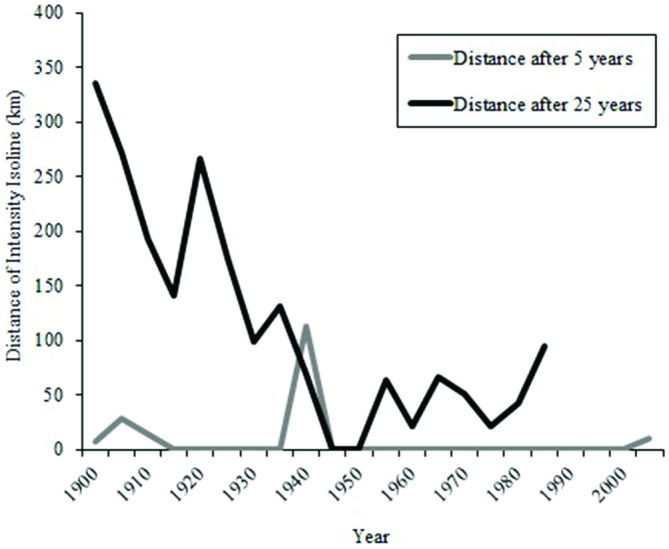
**Distances that an intensity isoline intersecting Ascension Island at a point in time moved during the next 5 and 25 years.** For example, the intensity isoline intersecting Ascension Island in the year 1900 moved approximately 5 km by 1905 but nearly 350 km by 1925.

During the early part of the century, the overlap between isolines and waterborne odorants for an odorant duration of 15 days was not as frequent as it was later in the century (Figure 8). The greatest amount of movement of the 25-year intensity isoline occurred prior to 1940 (Figure 9).

## Discussion

The results from the simulations and mapping provide evidence that a two-phase navigation strategy involving sequential use of magnetic and chemical cues is a plausible strategy for green turtles to use when migrating to Ascension Island. Based on known rates of magnetic field drift during the past century, as well as ocean currents and conditions in the South Atlantic, a strategy of moving along the intensity isoline that existed at Ascension during a turtle’s previous visit would usually (though not always) bring the turtle into contact with chemosensory cues associated with the island, regardless of whether the turtle is absent for 5 or 25 years (Figures 4, 6, 7). Thus, chemical cues might essentially enlarge the target area for turtles, allowing them to reach Ascension Island in one of two ways: (1) by exploiting a chemical plume originating at the island (Vickers, 2000; Moore and Crimaldi, 2004; Vasey et al., 2015); or (2) by using a search strategy to locate the island even if turtles are not able to travel directly to it (Lohmann et al., 1999; Akesson et al., 2003).

The chemical senses potentially available to turtles include olfaction, gustation, and vomeronasal chemoreception (Schwenk, 2008). Because sea turtles perceive chemical cues both in water and air (Manton et al., 1972; Grassman and Owens, 1982; Grassman et al., 1984; Endres et al., 2009; Endres and Lohmann, 2012, 2013), we ran simulations involving both airborne and waterborne chemical signals. We do not know if turtles depend more on one than the other, but regardless, a strategy that employed either or both in the latter stages of long-distance navigation would, in most cases, allow turtles to come into contact with Ascension Island after migrating into its proximity using magnetic information.

For waterborne chemical cues, a general principle that emerged from the simulations is that the outcome depends in part on the durability of the chemical: the longer the odorant remains in the environment, the better the multi-modal navigational mechanism performs. In the absence of any information on the nature of the chemical odorant or odorants that might originate at Ascension and be sensed by turtles, we can only speculate as to how long a possible chemical cue might persist in the surface waters of the equatorial Atlantic. For purposes of this initial analysis, we modeled scenarios in which the chemical(s) persist for 15, 30, and 45 days. A duration of less than 15 days would presumably diminish performance. By contrast, a duration longer than 45 days would have little or no effect on the outcome, given that odor plumes persisting for 45 days intersected all intensity isolines in all simulations (Figures 4, 5, 8).

For airborne cues, we arbitrarily assumed a duration of 48 h, while recognizing that it is impossible to predict with confidence how long unknown airborne odorants (or their degradation byproducts) are likely to persist in the environment. Because the wind consistently pushed particles directly in a WNW or NW direction, longer durations would presumably yield identical outcomes in most cases (because the windborne particles typically intersected magnetic isolines in less than 48 h). Likewise, slightly shorter durations are likely to yield similar results, whereas ephemeral odorants (e.g., those lasting only minutes) are unlikely to endure long enough to reach the isolines.

Previous tracking studies have yielded findings consistent with the idea that turtles might use olfactory or other local cues to locate Ascension Island once they are in its proximity. Nesting green turtles captured at the island and displaced to various locations offshore returned to the island after following circuitous routes, which might reflect a search for sensory cues associated with the target area (Luschi et al., 2001; Akesson et al., 2003). In addition to chemical cues emanating from the island or from nesting turtles, other mechanisms that might potentially help turtles pinpoint the location of a nearby island include visual cues (e.g., clouds accumulating near mountain tops), waves refracted around the island, and sounds of waves breaking (Lohmann et al., 1999, 2008a,c; Freake et al., 2006).

Contrary to earlier proposals (Koch et al., 1969; Carr, 1972), chemical cues emanating from Ascension Island seem unlikely to provide turtles with useful navigational information over most of their migration. Even if odorants from Ascension persist in the ocean for many months without degradation, it is difficult to imagine how an odor plume emanating from a source hundreds to thousands of kilometers away could remain sufficiently organized to reliably guide turtles across some 2000 km of open sea. Similarly, over most of the century, magnetic cues alone would not permit turtles to reach Ascension, particularly in the case of turtles returning to the island for the first time after 25 years (Figure 9; Lohmann et al., 1999; Freake et al., 2006). It is possible, however, that magnetic and chemical cues together might guide turtles to the island.

Although the analyses we have carried out focus on Ascension Island, the findings are likely to be applicable to a wide range of situations and geographic locations. A turtle navigating to any nesting beach or any specific foraging area, whether on an island or a mainland, might benefit from the use of multiple sensory cues. For turtles engaged in natal homing, in which adults return to reproduce in their area of origin after first migrating long distances away, the process might be facilitated if turtles imprint on both olfactory cues and magnetic cues that exist at their natal beach (Lohmann et al., 2008b, 2013; Putman and Lohmann, 2008). By doing so, a turtle might use the magnetic information on which it imprinted to arrive in the vicinity of the target area, and then employ chemical cues to locate a suitable place to nest. A similar process is thought to occur in Pacific salmon, which apparently use magnetic cues to navigate to the vicinity of their natal rivers and then use olfactory cues to identity the specific streams in which they hatched (Lohmann et al., 2008c; Putman et al., 2013). More broadly, multiple sensory sources are evidently used during long-distance migrations not only by marine animals (Lohmann et al., 2008c), but also by long-distance terrestrial migrants such as monarch butterflies (Reppert et al., 2010; Guerra et al., 2014) and migratory birds (Wiltschko and Wiltschko, 2003).

In sum, our modeling results provide evidence that a multi-modal approach to long-distance navigation is a plausible mechanism for turtles returning to Ascension Island and potentially, for those migrating long distances to other nesting and feeding areas. It is important to recognize, of course, that our findings do not demonstrate that this strategy is actually used by turtles navigating to remote islands; instead, our results indicate only that such a strategy is, in principle, feasible. Future studies will be needed to determine whether adult turtles migrating to Ascension or elsewhere do indeed rely on such a dual-cue strategy and if so, exactly how magnetic and chemical cues are used together in the process.

## Author Contributions

CSE contributed research and wrote the manuscript. NFP, CMFL, DAE, JAK, and KJL contributed research and edited drafts of the manuscript.

## Conflict of Interest Statement

The authors declare that the research was conducted in the absence of any commercial or financial relationships that could be construed as a potential conflict of interest.

## References

[B1] Akesson S. Broderick A. C. Glen F. Godley B. J. Luschi P. Papi F. (2003). Navigation by green sea turtles: which strategy do displaced adults use to find Ascension Island? Oikos 103, 363–372. 10.1034/j.1600-0706.2003.12207.x

[B2] Allard M. W. Miyamoto M. M. Bjorndal K. A. Bolten A. B. Bowen B. W. (1994). Support for natal homing in green turtles from mitochondrial DNA sequences. Copeia 1994, 34–41. 10.2307/1446668

[B3] Bleck R. (2002). An oceanic general circulation model framed in hybrid iopycnic-Cartesian coordinates. Ocean Model. 37, 55–88. 10.1016/s1463-5003(01)00012-9

[B4] Bracis C. Anderson J. J. (2012). An investigation of the geomagnetic imprinting hypothesis for salmon. Fish. Oceanogr. 21, 170–181. 10.1111/j.1365-2419.2012.00617.x

[B5] Brothers J. R. Lohmann K. J. (2015). Evidence for geomagnetic imprinting and magnetic navigation in the natal homing of sea turtles. Curr. Biol. 25, 392–396. 10.1016/j.cub.2014.12.035 25601546

[B7] Carr A. (1967). So Excellent a Fishe: A Natural History of Sea Turtles. New York: Natural History Press.

[B6] Carr A. (1972). “The case for long-range, chemoreceptive piloting in *Chelonia*,” in Animal Orientation and Navigation, eds Galler S. R. Schmidt-Koenig G. J. Jacobs R. E. Belleville R. E. (Washington, DC: NASA), 469–483.

[B8] Carr A. (1975). The Ascension Island green turtle colony. Copeia 3, 547–555. 10.2307/1443656

[B9] Chassignet E. P. Hurlburt H. E. Smedstad O. M. Halliwell G. R. Wallcraft A. J. Metzger E. J. (2006). Generalized vertical coordinates for eddy-resolving global and coastal ocean forecasts. Oceanography 19, 20–31. 10.5670/oceanog.2006.95

[B10] Constantino M. A. Salmon M. (2003). Role of chemical and visual cues in food recognition by leatherback posthatchlings (*Dermochelys coriacea* L). Zoology (Jena) 106, 173–181. 10.1078/0944-2006-00114 16351903

[B11] Cousins I. T. Mackay D. Parkerton T. F. (2003). “Physical-chemical properties and evaluative fate modelling of phthalate esters,” in The Handbook of Environmental Chemistry, (Vol. 3), part Q, ed. Staples C. (Berlin: Springer), 57–84.

[B12] Draxler R. R. (1999). HYSPLIT4 User’s Guide. *NOAA Tech. Memo*. ERL ARL-230, NOAA Air Resources Laboratory, Silver Spring, MD.

[B13] Draxler R. R. Hess G. D. (1997). Description of the HYSPLIT_4 Modeling System. NOAA Tech. Memo ERL ARL-224. NOAA Air Resources Laboratory, Silver Spring, MD.

[B14] Draxler R. R. Hess G. D. (1998). An overview of the HYSPLIT_4 modeling system of trajectories, dispersion, and deposition. Aust. Meteor. Mag. 47, 295–308.

[B17] Endres C. S. Lohmann K. J. (2012). Perception of dimethyl sulfide (DMS) by loggerhead sea turtles: a possible mechanism for locating high-productivity areas for foraging. J. Exp. Biol. 215, 3535–3538. 10.1242/jeb.073221 23014568

[B15] Endres C. S. Lohmann K. J. (2013). Detection of coastal mud odors by loggerhead sea turtles: a possible mechanism for sensing nearby land. Mar. Biol. 160, 2951–2956. 10.1007/s00227-013-2285-6

[B16] Endres C. S. Putman N. F. Lohmann K. J. (2009). Perception of airborne odors by loggerhead sea turtles. J. Exp. Biol. 212, 3823–3827. 10.1242/jeb.033068 19915124

[B18] Frazer N. B. Ehrhart L. M. (1985). Preliminary growth models for green, *Chelonia mydas* and loggerhead, *Caretta caretta*, turtles in the wild. Copeia 1985, 73–79. 10.2307/1444792

[B19] Frazer N. B. Ladner R. C. (1986). A growth curve for green sea turtles, *Chelonia mydas*, in the U.S. Virgin Islands, 1913–1914. Copeia 1986, 798–802. 10.2307/1444963

[B20] Freake M. J. Muheim R. Philips J. B. (2006). Magnetic maps in animals: a theory comes of age? Q. Rev. Biol. 81, 327–347. 10.1086/511528 17240727

[B21] Godley B. J. Broderick A. C. Hays G. C. (2001). Nesting of green turtles (*Chelonia mydas*) at Ascension Island, South Atlantic. Biol. Conserv. 97, 151–158. 10.1016/s0006-3207(00)00107-5

[B22] Gould J. L. (1982). The map sense of pigeons. Nature 296, 205–211. 10.1038/296205a0

[B23] Grassman M. A. Owens D. W. (1982). Development and extinction of food preferences in the loggerhead sea turtles, *Caretta caretta*. Copeia 1982, 965–969. 10.2307/1444110

[B24] Grassman M. A. Owens D. W. McVey J. P. Marquez M. (1984). Olfactory-based orientation in artificially imprinted sea turtles. Science 224, 83–84. 10.1126/science.224.4644.83 17783530

[B25] Guerra P. A. Gegear R. J. Reppert S. M. (2014). A magnetic compass aids monarch butterfly migration. Nat. Commun. 5:4164. 10.1038/ncomms5164 24960099 PMC4090716

[B26] Harden-Jones F. R. (1968). Fish Migration. New York: St. Martin’s Press.

[B27] Hays G. C. Akesson S. Broderick A. C. Glen F. Godley B. J. Luschi P.. (2001). The diving behavior of green turtles undertaking oceanic migration to and from Ascension Island: dive durations, dive profiles and depth distribution. J. Exp. Biol. 204, 4093–4098. 11809783 10.1242/jeb.204.23.4093

[B28] Hoffman J. I. Forcada J. (2012). Extreme natal philopatry in female Antarctic fur seals (*Arctocephalus gazelle*). Mammal Biol. 77, 71–73. 10.1016/j.mambio.2011.09.002

[B29] Koch A. L. Carr A. Ehrenfeld D. W. (1969). The problem of open-sea navigation: the migration of the green turtles to Ascension Island. J. Theor. Biol. 22, 163–179. 10.1016/0022-5193(69)90085-x 5797566

[B30] Lett C. Verley P. Mullon C. Parada C. Brochier T. Penven P. (2008). A Lagrangian tool for modeling ichthyoplankton dynamics. Environ. Model. Softw. 23, 1210–1214. 10.1016/j.envsoft.2008.02.005

[B31] Lohmann K. J. Lohmann C. M. F. (1994). Detection of magnetic inclination angle by sea turtles: a possible mechanism for determining latitude. J. Exp. Biol. 194, 23–32. 9317267 10.1242/jeb.194.1.23

[B32] Lohmann K. J. Lohmann C. M. F. (1996). Detection of magnetic field intensity by sea turtles. Nature 380, 59–61. 10.1038/380059a0

[B33] Lohmann K. J. Hester J. T. Lohmann C. M. F. (1999). Long-distance navigation in sea turtles. Ethol. Ecol. Evol. 11, 1–23. 10.1080/08927014.1999.9522838

[B35] Lohmann K. J. Lohmann C. M. F. Putman N. F. (2007). Magnetic maps in animals: nature’s GPS. J. Exp. Biol. 210, 3697–3705. 10.1242/jeb.001313 17951410

[B36] Lohmann K. J. Lohmann C. M. F. Ehrhart L. M. Bagley D. A. Swing T. (2004). Geomagnetic map used in sea turtle navigation. Nature 428, 909–910. 10.1038/428909a 15118716

[B37] Lohmann K. J. Lohmann C. M. F. Rogers J. R. Putman N. F. (2013). “Natal homing and imprinting in sea turtles,” In Biology of Sea Turtles, (Vol. 3), eds Wyneken J. Lohmann K. J. Musick J. (Boca Raton, FL: CRC Press), 59–77.

[B38] Lohmann K. J. Luschi P. Hays G. C. (2008a). Goal navigation and island-finding in sea turtles. J. Exp. Mar. Biol. Ecol. 356, 83–95. 10.1016/j.jembe.2007.12.017

[B40] Lohmann K. J. Putman N. F. Lohmann C. M. F. (2008b). Geomagnetic imprinting: a unifying hypothesis of long-distance natal homing in salmon and sea turtles. Proc. Nat. Acad. Sci. U S A 105, 19096–19101. 10.1073/pnas.0801859105 19060188 PMC2614721

[B34] Lohmann K. J. Lohmann C. M. F. Endres C. S. (2008c). The sensory ecology of ocean navigation. J. Exp. Biol. 211, 1719–1728. 10.1242/jeb.015792 18490387

[B39] Lohmann K. J. Putman N. F. Lohmann C. M. F. (2012). The magnetic map of hatchling loggerhead sea turtles. Curr. Opin. Neurobiol. 22, 336–342. 10.1016/j.conb.2011.11.005 22137566

[B41] Luschi P. Akesson S. Broderick A. C. Glen F. Godley B. J. Papi F. (2001). Testing the navigational abilities of ocean migrants: displacement experiments on green sea turtles (*Chelonia mydas*). Behav. Ecol. Sociobiol. 50, 528–534. 10.1007/s002650100396

[B42] MacMillan S. Maus S. (2005). International geomagnetic reference field - the tenth generation. Earth Planets Space 57, 1135–1140. 10.1186/bf03351896

[B43] Manton M. Karr A. Ehrenfeld D. W. (1972). Chemoreception in the migratory sea turtle, *Chelonia mydas*. Biol. Bull. 143, 184–195. 10.2307/1540338 4653343

[B44] Moore P. Crimaldi J. (2004). Odor landscapes and animal behavior: tracking odor plumes in different physical worlds. J. Mar. Sys. 49, 55–64. 10.1016/j.jmarsys.2003.05.005

[B45] Mortimer J. A. Carr A. (1987). Reproduction and migrations of the Ascension island green turtle. Copeia 1, 103–113. 10.2307/1446043

[B46] Mortimer J. A. Poirtier K. M. (1989). Reproductive homing and internesting behavior of the green turtle (*Chelonia mydas*) at Ascension Island, South Atlantic Ocean. Copeia 4, 962–977. 10.2307/1445982

[B47] Piovano S. Balletto E. Di Marco S. Dominici A. Giacoma C. Zanetti A. (2004). Loggerhead turtle (*Caretta caretta*) bycatches on long-lines: the importance of olfactory stimuli. Ital. J. Zool. 71, 213–216. 10.1080/11250000409356638

[B52] Putman N. F. Endres C. S. Lohmann C. M. F. Lohmann K. J. (2011). Longitude perception and bicoordinate magnetic maps in sea turtles. Curr. Biol. 21, 463–466. 10.1016/j.cub.2011.01.057 21353561

[B50] Putman N. F. He R. (2013). Tracking the long-distance dispersal of marine organisms: sensitivity to ocean model resolution. J. R. Soc. Interface 10:20120979. 10.1098/rsif.2012.0979 23349437 PMC3627105

[B49] Putman N. F. Jenkins E. S. Michielsens C. G. J. Noakes D. L. G. (2014). Geomagnetic imprinting predicts spatiotemporal variation in homing migration of pink and sockeye salmon. J. R. Soc. Interface 11:20140542. 10.1098/rsif.2014.0542 25056214 PMC4233730

[B48] Putman N. F. Lohmann K. J. (2008). Compatibility of magnetic imprinting and secular variation. Curr. Biol. 18, R596–R597. 10.1016/j.cub.2008.05.008 18644331

[B53] Putman N. F. Lohmann K. J. Putman E. M. Klimley A. P. Quinn T. P. Noakes D. L. G. (2013). Evidence for geomagnetic imprinting as a homing mechanism in Pacific salmon. Curr. Biol. 23, 312–316. 10.1016/j.cub.2012.12.041 23394828

[B51] Putman N. F. Mansfield K. L. (2015). Direct evidence of swimming demonstrates active dispersal in the sea turtle “lost years”. Curr. Biol. 25, 1221–1227. 10.1016/j.cub.2015.03.014 25866396

[B54] Reppert S. M. Gegear R. J. Merlin C. (2010). Navigational mechanisms of migrating monarch butterflies. Trends Neurosci. 33, 399–406. 10.1016/j.tins.2010.04.004 20627420 PMC2929297

[B55] Rooker J. R. Secor D. H. De Metrio G. Schloesser R. Block B. A. Neilson J. D. (2008). Natal homing and connectivity in Atlantic bluefin tuna populations. Science 31, 742–744. 10.1126/science.1161473 18832611

[B56] Schwenk K. (2008). “Comparative anatomy and physiology of chemical senses in nonavian aquatic reptiles,” in Sensory Evolution on the Threshold. Adaptations in Secondarily Aquatic Vertebrates, eds Thewissen J. G. M. Nummela S. (Berkeley: University of California Press), 65–81.

[B57] Skiles D. D. (1985). “Geomagnetic field, its nature, history and significance in biology,” in Magnetite Biomineralization and Magnetoreception in Organisms, eds Kirschvink J. L. Jones D. S. McFadden B. J. (New York: Plenum Press), 43–102.

[B58] Svedäng H. Righton D. Jonsson P. (2007). Migratory behavior of Atlantic cod *Gadus morhua*: natal homing is the prime stock-separating mechanism. Mar. Ecol. Prog. Ser. 345, 1–12. 10.3354/meps07140

[B59] Vasey G. Lukeman R. Wyeth R. C. (2015). Additional navigational strategies can augment odor-gated rheotaxis for navigation under conditions of variable flow. Integr. Comp. Biol. 55, 447–460. 10.1093/icb/icv073 26116202

[B60] Vickers N. J. (2000). Mechanisms of animal navigation in odor plumes. Biol. Bull. 198, 203–212. 10.2307/1542524 10786941

[B61] Wiltschko R. Wiltschko W. (2003). Avian navigation: from historical to modern concepts. Anim. Behav. 65, 257–272. 10.1006/anbe.2003.2054

